# Identification and management of asymptomatic carriers of coronavirus disease 2019 (COVID‐19) in China

**DOI:** 10.1111/irv.12768

**Published:** 2020-06-08

**Authors:** Jianhui Peng, Dongwei Su, Ziwei Zhang, Mingke Wang

**Affiliations:** ^1^ Department of Quality Management Guangdong Second Provincial General Hospital Guangzhou China; ^2^ Department of Thyroid and Breast Surgery Changhai Hospital Naval Medical University Shanghai China; ^3^ School of Life Science Sun Yat‐Sen University Guangzhou China; ^4^ Naval Special Medical Center Naval Medical University Shanghai China; ^5^ Department of Epidemic Prevention No. 92371 Unit Hospital of PLA Fujian China

**Keywords:** asymptomatic carriers, COVID‐19, identification, management, SARS‐CoV‐2

To the Editor,

We read with interest the recent article by Yu et al, which reported asymptomatic transmission of coronavirus disease 2019 (COVID‐19).^1^ Asymptomatic carriers of COVID‐19 who have no clinical symptoms, but test positive for the virus that causes COVID‐19 virus (SARS‐CoV‐2) in respiratory specimens or other specimens, have attracted the attention of scientists all over the world.^2^, ^3^ COVID‐19 transmission through asymptomatic carriers has been a huge challenge for COVID‐19 prevention and control.^1^ There are two types of asymptomatic carriers: those who never develop symptoms and those who are detected during the incubation period (pre‐symptomatic detection) prior to symptom onset.^3^ Here, we discussed the identification and management of asymptomatic carriers of COVID‐19 in China.

So far, asymptomatic carriers of COVID‐19 may be found by the following ways.^3^ First, close contacts of confirmed cases of COVID‐19 may be identified as asymptomatic carriers during their medical observation period. Second, asymptomatic carriers have been found in the investigation of cluster outbreaks of COVID‐19 by active detection. Third, asymptomatic infectors can be detected when tracing the potential infection sources of COVID‐19 patients. Fourth, asymptomatic carriers may be detected in the screening populations with a history of travel or living in epidemic areas of COVID‐19. Fifth, asymptomatic carriers could be detected during epidemiological investigations and opportunistic screening. Previous studies have reported asymptomatic transmission to family members^4^, ^5^ and described an example of asymptomatic transmission during the incubation period.^6^ Asymptomatic carriers can shed similar amounts of virus to symptomatic patients.^7^

Since April 1, 2020, the number of asymptomatic infectors has been reported online by National Health Commission of the People's Republic of China on a daily basis, and the following management measures were required to be carried out to minimize the risk of their transmission in China.^3^ First, persons detected as asymptomatic infectors would be isolated for 14 days, which may prevent them from becoming contagion sources. Those can be lifted from isolation by negative nucleic acid tests on two consecutive samples at least 24 hours apart.^3^ Second, epidemiological investigation of asymptomatic infectors would be strengthened, and strict disinfection would be implement in their living places such as homes, medical institutions, isolation wards, transport tools, and medical observation places. Third, since early detection of asymptomatic carriers is critical to contain their transmission, current screening methods also need to be strengthened. Nucleic acid screening is practical and quick for the population. However, due to specimen collection, testing methods, product stability, false‐negative results have been frequently reported, which will hamper case detection and disease control.^8^ Therefore, multiple screening and monitoring of nucleic acid combining with antigens and antibodies in blood and other body fluids are recommended.^8^

At present, persons who are significant epidemiological associations with COVID‐19 patients (eg, close contacts) will be put under 14‐day centralized medical observation in China.^3^ As the epidemic enters a new stage, in order to consolidate the previous anti‐epidemic achievements and prevent the epidemic from rebounding, we should further strengthen the monitoring of asymptomatic carriers and some special populations who may play a greater role in the spread of COVID‐19, including front‐line medical staff, disease control personnel, street epidemic prevention and control point staff, and delivery personnel. Since asymptomatic carriers have no clinical symptoms, they are difficult to identify, diagnose, and isolate. This can lead to loopholes in prevention and control measures, resulting in increased difficulty in controlling the spread of COVID‐19.^9^ The public health education should be strengthened, and formation of good hygiene habits is important. In particular, awareness of self‐protection, self‐supervision and administration, and pre‐service training of above special populations are critical to reducing the spread of asymptomatic infections.

In future, further definition of high‐risk populations and development of effective screening strategies and programs will support rapid identification and management of asymptomatic carrier transmission of COVID‐19.^9^ Further study is needed on asymptomatic carriers including their frequency relative to symptomatic infections, their disease course, and factors associated with having an asymptomatic rather than symptomatic infection.^8^ Since there can be a gray area between asymptomatic and symptomatic infections, we also need to improve the detection of infections with very mild subclinical disease who may not seek medical attention but may also be responsible for transmission in the community. With so many scientific questions to be addressed in asymptomatic carriers of COVID‐19, canceling public gatherings, implementing strong social‐distancing measures, washing your hands, and wearing a mask may be the best way to stop the virus from spreading.

In conclusion, asymptomatic carriers of COVID‐19 can be contagious. Identification and management of these asymptomatic infectors has been strengthened in China. These measures may also help other countries to combat the COVID‐19 epidemic.

## CONFLICT OF INTEREST

The authors declare that they have no competing interests.

## AUTHOR CONTRIBUTION

**Jianhui Peng:** Formal analysis; Writing‐original draft; Writing‐review & editing (equal). **Dongwei Su:** Formal analysis; Writing‐original draft; Writing‐review & editing (equal). **Ziwei Zhang:** Writing‐review & editing (equal). **Mingke Wang:** Conceptualization (lead); Formal analysis (equal); Project administration (lead); Supervision (lead); Validation (lead); Writing‐original draft (equal); Writing‐review & editing (lead).

## ETHICAL STATEMENT

The article does not contain the participation of any human being and animal.
